# The Chemical Composition and Dietetic Value of Wines

**Published:** 1907-06-15

**Authors:** 


### The Hospital

A Journal of

The Medical Sciences and Hospital Administration.

New Series. No. 12, Vol. I. [No. 1084, Vol. XLII.] SATURDAY, JUNE 15, 1907.

### The Chemical Composition and Dietetic Value of Wines.

With this issue we present to our readers a special report on the chemical composition and dietetic value of wines. The report embodies the results of a prolonged and elaborate laboratory investigation instituted for the purpose of obtaining a secure and confident knowledge of the effects produced on the physiological functions of the body by various alcoholic beverages. Many of these beverages are widely used in domestic and social life, and their employment has frequently been attacked, and as frequently defended. It is beyond question that used in excess they are capable of producing much harm, not only in the shape of disturbed function, but also in the form of gross and serious pathological changes. But to state this is not to close the discussion in reference to their use in moderation. Upon this latter point appeal is frequently and naturally made to the medical profession. In the light of recent history it cannot be said that the profession, or at least certain members of it, have been slow to provide an answer. Unfortunately, however, the answers supplied have often been indirectly conflicting. For this doubtless many reasons may be suggested. But one of these most certainly is the absence of positive knowledge based upon observations capable of scientific demonstration and critical verification. On the one hand, are the disastrous moral and pathological effects produced by excess. On the other, the testimony afforded by the widespread customs and habits of mankind. One group of medical authorities, contemplating more particularly the former of these two facts, denounces the use of alcohol in every shape and form; while their opponents quote in reply the general verdict of those who practise moderation. So long as the issue is left in this position no progress is possible. It is necessary to transfer the debate to an arena where there is no room for personal feeling or heated declamation, and where the verdict shall be decided by facts, as these are established by impartial and scientific standards. These conditions are supplied by the accurate and precise methods of the laboratory, and it is on an authority of this order that the conclusions of our report rest. It is further necessary to remind our readers that the discussion relates not simply and solely to alcohol, but to alcoholic beverages. Only when this is recognised does the question of laboratory investigation come into contact with the actual facts of life. The use of pure alcohol as a beverage need hardly be considered, for it has no practical application. The various drinks more or less widely employed, alike in civilised and uncivilised communities, are the products of fermentation, and the chemical process which produces alcohol produces at the same time many other substances. It is to the latter, indeed, that the palate-pleasing properties of many of these beverages are due. From all this it is evident that a mere recital of the virtues or vices of alcohol does not exhaust the issue. It is necessary to deal with the beverages in the form and quality in which they are actually employed. Especially is this to be recognised in a discussion which is concerned with such highly complex fluids as wines. In many of these, as our report shows, the alcoholic content is a character of entirely secondary importance. It is the so-called by-products which confer individuality on the various classes of wines, and the use of these latter as beverages demands, therefore, an appreciation of the physiological values and actions of their non-alcoholic constituents. Particularly in this respect would we draw attention to the distinctions which exist between wines and spirits. In each by-products exist, but those of the one group are quite different from those of the other. These facts are sufficient in themselves to show that neither an undiscriminating recommendation nor a wholesale condemnation of alcoholic beverages can be justified. The term covers substances which differ widely both in their chemical composition and their physiological actions. It is necessary, therefore, to recognise these differences, and to deal with the situation as the facts demand. Towards that end we need not hesitate to say the investigations on which our report is founded offer a real and substantial contribution.

Whilst doubtless interested in the general question, the great majority of our readers will desire specific information in reference to the use of alcoholic beverages in the treatment of disease. And in this direction there is much to be learned from the facts and conclusions set forth in the report. Here, more particularly, is recognition of essential quantitative and qualitative variations and effects. What is true of the contrasted positions of wines and spirits is equally true as regards different classes of wines. The specific qualities and actions which differentiate these from one another do not depend merely on the percentages of alcohol they severally contain. The non-alcoholic constituents have also their own special range and function. Consequently, if alcoholic beverages are to be prescribed appropriately and with intelligence, a full analysis must be appreciated, and a selection must be exercised founded on knowledge of the wants of the individual case. The choice, indeed, must be made, if success is to be obtained, upon a similar basis to that on which rests the choice of medicinal agents generally. We claim here to set before our readers a series of careful and decisive pharmacological experiments which may be used as guides to the therapeutic use of wines in the treatment of disease. Specific and detailed directions are as necessary when prescribing these as they are when using ordinary medicinal agents. Altogether we feel sure that a study of the report will prove an aid to our readers in a department of opinion and practice which has too long been the sport of vague impressions and confident but unsupported statements.

